# The Atad5 RFC-like complex is the major unloader of proliferating cell nuclear antigen in *Xenopus* egg extracts

**DOI:** 10.1016/j.jbc.2023.105588

**Published:** 2023-12-21

**Authors:** Yoshitaka Kawasoe, Sakiko Shimokawa, Peter J. Gillespie, J. Julian Blow, Toshiki Tsurimoto, Tatsuro S. Takahashi

**Affiliations:** 1 Faculty of Science, Kyushu University, Fukuoka, Japan; 2 Graduate School of Systems Life Sciences, Kyushu University, Fukuoka, Japan; 3 Division of Molecular, Cell & Developmental Biology, School of Life Sciences, University of Dundee, Dundee, UK

**Keywords:** proliferating cell nuclear antigen, replication clamp, clamp loader, *Xenopus* egg extract, DNA replication, mismatch repair

## Abstract

Proliferating cell nuclear antigen (PCNA) is a homo-trimeric clamp complex that serves as the molecular hub for various DNA transactions, including DNA synthesis and post-replicative mismatch repair. Its timely loading and unloading are critical for genome stability. PCNA loading is catalyzed by Replication factor C (RFC) and the Ctf18 RFC-like complex (Ctf18-RLC), and its unloading is catalyzed by Atad5/Elg1-RLC. However, RFC, Ctf18-RLC, and even some subcomplexes of their shared subunits are capable of unloading PCNA *in vitro*, leaving an ambiguity in the division of labor in eukaryotic clamp dynamics. By using a system that specifically detects PCNA unloading, we show here that Atad5-RLC, which accounts for only approximately 3% of RFC/RLCs, nevertheless provides the major PCNA unloading activity in *Xenopus* egg extracts. RFC and Ctf18-RLC each account for approximately 40% of RFC/RLCs, while immunodepletion of neither Rfc1 nor Ctf18 detectably affects the rate of PCNA unloading in our system. PCNA unloading is dependent on the ATP-binding motif of Atad5, independent of nicks on DNA and chromatin assembly, and inhibited effectively by PCNA-interacting peptides. These results support a model in which Atad5-RLC preferentially unloads DNA-bound PCNA molecules that are free from their interactors.

Eukaryotic chromosome replication is a complex process involving DNA synthesis, Okazaki fragment maturation, chromatin assembly, and post-replicative mismatch repair (MMR) (1, 2). These processes are tightly regulated to occur in the right order at the right place. The replication clamp, a ring-shaped protein complex structurally conserved among three domains of life, encircles DNA, translocates along DNA, and interacts with numerous factors related to replication and repair (3, 4). Its loading and unloading are critical steps for the sequential and temporal regulation of the assembly and disassembly of multiprotein complexes required for various DNA transactions.

The eukaryotic replication clamp is proliferating cell nuclear antigen (PCNA), a homo-trimeric complex whose monomer consists of two structurally similar N- and C-globular domains connected by the interdomain connecting loop. The interaction between PCNA and its binding partners is frequently mediated through a short peptide that binds to a hydrophobic pocket formed underneath the interdomain connecting loop. A major class of such short peptides is the PCNA-interacting peptide (PIP) box (5, 6), an octapeptide motif found in many PCNA interactors, including DNA polymerases, Flap structure-specific endonuclease 1 (Fen1), DNA ligase 1 (Lig1), Chromatin assembly factor 1 (CAF-1), and the MutSα (Msh2–Msh6) and MutSβ (Msh2–Msh3) mismatch sensor complexes (3, 4). Through the interaction with its binding partners, PCNA coordinates multiple reactions of chromatin replication. During lagging strand synthesis, for example, PCNA recruits DNA polymerase δ (Pol δ) onto short primers synthesized by DNA polymerase α (Pol α) to extend Okazaki fragments, Fen1 and Lig1 to process and ligate Okazaki fragments, and CAF-1 to assemble chromatin on the nascent DNA (1, 3, 4). During and after DNA synthesis, PCNA also plays multiple critical roles in MMR: It recruits MutSα and MutSβ mismatch sensors to the replication factory, activates the MutLα nicking endonuclease in a strand-specific manner, thereby functioning as a marker for the newly-synthesized DNA strand, and supports repair DNA synthesis by Pol δ (7, 8, 9, 10, 11, 12, 13, 14, 15, 16, 17, 18). Eukaryotes also have another structurally related clamp complex, Rad9–Hus1–Rad1 (9–1–1), a hetero-trimeric complex dedicated to DNA damage checkpoint activation and repair (19).

Since free PCNA forms a closed ring structure, its DNA loading must involve the opening of the ring. This process is catalyzed by the evolutionarily conserved clamp loader complexes (19, 20, 21, 22). The major eukaryotic clamp loader, Replication Factor C (RFC), is a hetero-pentameric complex of Rfc1–5, all of which share the AAA+ ATPase domain and are arranged in a spiral conformation (23, 24). RFC has four active ATP-binding sites, and a series of studies demonstrate that sequential ATP binding to RFC induces RFC–PCNA complex formation, the spring-washer-like opening of the PCNA ring, and the binding of the RFC–PCNA complex onto the 3′-terminus of a primer-template junction (24, 25, 26, 27). Subsequent hydrolysis of ATP molecules facilitates the closure of the PCNA ring and the dissociation of RFC from PCNA (28).

Eukaryotes have three Rfc1 paralogs: Ctf18, Elg1 (ATAD5 in humans and Atad5 in *Xenopus*), and Rad17 (29, 30, 31, 32, 33, 34). These paralogs replace the largest Rfc1 subunit of RFC to form alternative RFC-like complexes (RLCs). Two additional subunits, Ctf8 and Dcc1, are also included in Ctf18-RLC (30, 35). Unlike other RFC/RLCs, Rad17-RLC is specialized for the loading of the 9–1–1 checkpoint clamp onto the single-stranded DNA region at the 5′-terminus of a junction between single-stranded and double-stranded DNA (36, 37, 38). Ctf18-RLC has PCNA-loading activity and forms an active PCNA-loading complex with the major leading-strand replicase DNA polymerase ε (Pol ε), and thus is proposed to be an alternative PCNA loader for the leading strand (39, 40, 41, 42, 43). Ctf18-RLC also promotes the establishment of sister chromatid cohesion (29, 30, 42). In contrast to RFC and Ctf18-RLC, no PCNA loading activity has been reported for Elg1/ATAD5-RLC. Rather, this complex is proposed to be a PCNA unloader, since deletion of *elg1* or depletion of its mammalian homolog ATAD5 leads to the accumulation of chromatin-bound PCNA, and purified Elg1-RLC and ATAD5-RLC exhibit PCNA unloading activity (44, 45, 46, 47). Deficiency of the *elg1*/*Atad5* gene in yeast and mice leads to chromosome instability, whilst also predisposing mice to cancer, suggesting that the timely unloading of PCNA is critical for genome stability (31, 32, 33, 48, 49). The regulation of PCNA unloading is also vital to MMR: MutSα interacts with PCNA and prevents its unloading to retain MMR capability (9, 13), while conversely, over-accumulation of PCNA on chromatin interferes with MMR (50).

Although evidence that Elg1/ATAD5-RLC is a primary PCNA unloader *in vivo* has been accumulated, PCNA unloading activity is clearly not specific to this complex. RFC is a pentameric complex of Rfc1, Rfc4, Rfc3, Rfc2, and Rfc5 arranged in this order, and Rfc1 is not essential for the opening of PCNA (51). Consistently, the human RFC4–RFC3–RFC2, the yeast Rfc4–Rfc3–Rfc2–Rfc5, and even the yeast Rfc2–Rfc5 subcomplexes are capable of unloading PCNA from nicked circular DNA, suggesting that the intrinsic PCNA unloading activity resides in the smaller subunits shared by all the clamp-loader/unloader complexes (51, 52). In agreement with this notion, PCNA unloading activity has been detected in all RFC and RLCs. In yeast, both RFC and Rad24-RLC (the yeast counterpart of Rad17-RLC) unload PCNA from singly-nicked circular DNA *in vitro* (51), and human RFC unloads PCNA from nicked circular DNA in a purified system and covalently closed DNA after replication by the SV40 system (53, 54). Likewise, yeast Ctf18-RLC unloads PCNA *in vitro* in the presence of replication protein A (RPA), a eukaryotic single-stranded DNA binding protein (55). These biochemical data raise the possibility that not only Elg1/ATAD5-RLC but also RFC, Ctf18-RLC, and Rad17-RLC contribute to PCNA unloading *in vivo*. However, the division of labor of RFC and other RLCs in PCNA unloading has not been fully determined, partly because the inactivation of clamp loaders masks their possible contribution to PCNA unloading by inhibiting the PCNA loading reaction.

To clarify the contribution of individual clamp loaders to PCNA unloading, we herein used an assay that specifically detects PCNA unloading in extracts prepared from the eggs of the frog *Xenopus laevis*, a physiological cell-free *in vitro* model system that recapitulates various DNA transactions, including PCNA loading/unloading and mismatch repair. Our data pinpoint Atad5-RLC as the major PCNA unloader in *Xenopus* egg extracts and support a model in which PCNA molecules that are free from their binding partners are preferentially targeted to be unloaded by Atad5-RLC.

## Results

**Figure 1:**
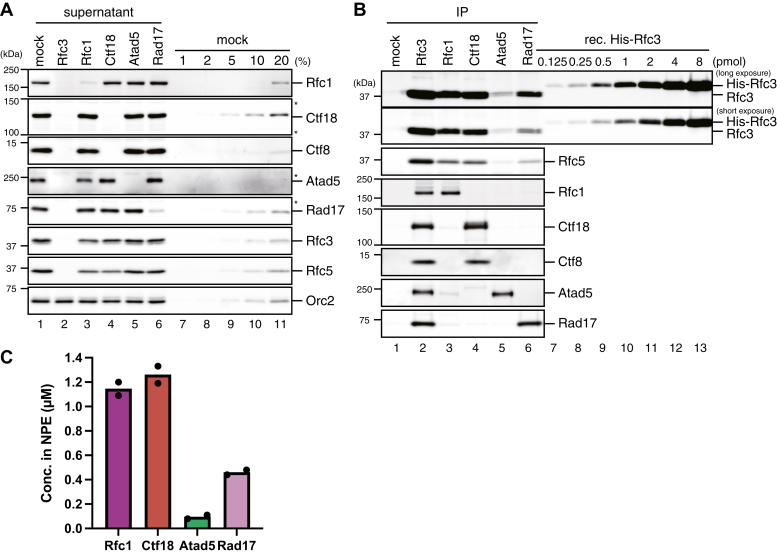
**Quantification of the concentration of RFC and RLCs in NPE.***A* and *B*, immunoprecipitation was performed from NPE using preimmune- (“mock”, lane 1), Rfc3- (lane 2), Rfc1- (lane 3), Ctf18- (lane 4), Atad5- (lane 5), or Rad17-antibodies (lane 6), and 0.25 μl each of the supernatant (*A*) and the IP fractions corresponding to 2.5 μl each of NPE (*B*) were separated by SDS-PAGE alongside a serial dilution series of the supernatant of mock-IP NPE (*A*) or the indicated amounts of recombinant His_6_-Rfc3 (*B*), followed by immunoblotting with indicated antibodies. Orc2 serves as a loading control. (∗) Cross-reacting band. *C*, the estimated concentrations of RFC and RLCs in NPE were plotted on a graph. Filled circles represent individual data from two independent experiments, including the one shown in (*B*).

The nucleoplasmic extract (NPE) of *Xenopus* eggs is a nearly undiluted extract of nuclear proteins and functionally recapitulates various DNA transactions, including DNA replication, PCNA loading and unloading, and mismatch repair (13, 56, 57). To gain insight into the division of labor of clamp loaders, we first quantified the concentration of RFC and RLCs in NPE. Immunoprecipitation (IP) of a shared subunit Rfc3 removed most of the Rfc1, Ctf18, Atad5, and Rad17 proteins from NPE, suggesting that the majority of the large subunits are included in Rfc3-containing complexes (Fig. 1*A*, lane 2). Consistently, simultaneous IP of Rfc1, Ctf18, Atad5, and Rad17 co-depleted more than 90% of Rfc3 and Rfc5 from NPE, suggesting that most Rfc3 and Rfc5 are included in the RFC/RLC complexes (Fig. S1*A*). Therefore, we reasoned that the large and small RFC/RLC subunits form stoichiometric complexes in NPE. Using recombinant His-tagged Rfc3 purified from bacteria, we quantified the amount of Rfc3 in the Rfc1-IP fraction, and based on this number, we then estimated the concentration of RFC in NPE at approximately 1.1 μM. Similarly, we estimated the concentrations of Ctf18-RLC, Atad5-RLC, and Rad17-RLC at 1.3, 0.10, and 0.46 μM, respectively (Figs. 1 and S1*B*).

**Figure 2:**
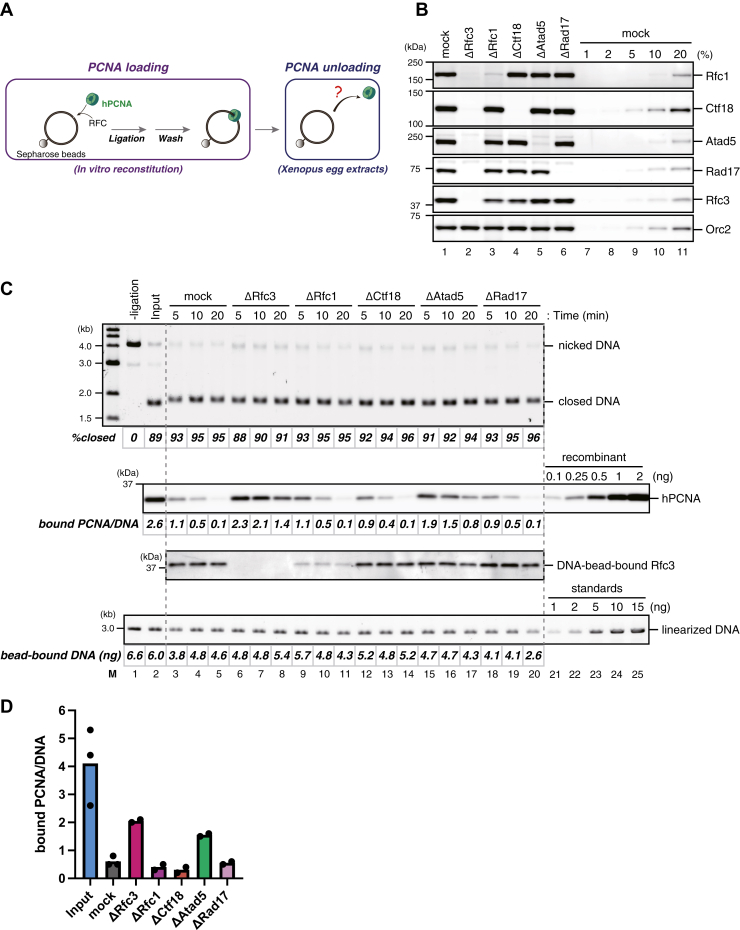
**Atad5-RLC is the major PCNA unloader in *Xenopus* egg extracts.***A*, a schematic diagram of the PCNA unloading assay. Singly biotinylated plasmid carrying a nick was bound to Sepharose beads and incubated with recombinant human PCNA and RFC to load PCNA on DNA. After nick ligation and extensive wash, the resulting PCNA-DNA complex was transferred into *Xenopus* egg extracts to examine PCNA unloading. *B*, 0.25 μl each of mock-treated (lane 1), Rfc3-depleted (lane 2), Rfc1-depleted (lane 3), Ctf18-depleted (lane 4), Atad5-depleted (lane 5), Rad17-depleted NPE (lane 6), and a serial dilution series of mock-treated NPE (lanes 7–11) were analyzed by immunoblotting with indicated antibodies. Orc2 serves as a loading control. *C*, PCNA loaded onto immobilized DNA was incubated in NPE described in (*B*). Untreated DNA separated by agarose gel electrophoresis in the presence of ethidium bromide, a quantitative immunoblot of hPCNA and Rfc3 in the bead-bound fractions, and linearized DNA (treated with XmnI) separated by agarose gel electrophoresis are presented along with the percentage of covalently-closed plasmids, the estimated numbers of DNA-bound PCNA molecules per plasmid, and the amount of bead-bound DNA. *D*, the average numbers of DNA-bound PCNA per plasmid at the 10-min time points were plotted into a graph. Filled circles represent individual data from two independent experiments, including the one shown in (*C*), except for the ‘Input’ and ‘mock’ samples, which were triplicated.

Biochemical studies with purified proteins have shown that not only Elg1/ATAD5-RLC but also RFC, Rad17-RLC, Ctf18-RLC, and several Rfc2–5 subcomplexes can unload PCNA from DNA (51, 52, 53, 54, 55). Given that Atad5-RLC represents only a minor fraction of total RFC/RLCs in *Xenopus* egg extracts, we next compared the PCNA unloading capability of all four RFC/RLC complexes. We previously established an assay that specifically detects PCNA unloading in *Xenopus* egg extracts (13). Briefly, we load human PCNA (hPCNA) with human RFC, 2 to 10 trimers per DNA on average, onto nicked-circular DNA immobilized on Sepharose beads, ligate the nick, wash out unbound proteins including RFC and DNA ligase, and incubate the hPCNA-DNA complex in the extracts (Fig. 2*A*). Since hPCNA can functionally replace xPCNA in NPE but is specifically detected with a monoclonal antibody that does not recognize *Xenopus* PCNA (xPCNA), this system can discriminate the unloading events of hPCNA from the possible loading events of xPCNA. In a mock-treated NPE, hPCNA was quickly unloaded from covalently closed circular DNA within 20 min (Fig. 2, *B* and *C*). Rfc3 was recovered in the DNA-bead fraction, likely reflecting the DNA-binding affinity of RFC/RLCs. In contrast, in the Rfc3-depleted NPE, where no Rfc3 binding was observed, the rate of the unloading of hPCNA was significantly retarded, suggesting that most of the unloading events in NPE depend on the Rfc3-containing complexes. Although we observed some hPCNA dissociation in Rfc3-depleted NPE, this level of PCNA dissociation likely reflects the intrinsic instability of hPCNA under our experimental condition since a similar level of hPCNA dissociation was observed in heat-inactivated *Xenopus* egg extracts (Fig. S2, *A* and *B*). Importantly, Atad5-depletion slowed down the rate of hPCNA unloading to a similar level to that seen in Rfc3-depleted NPE (Figs. 2, *B*–*D*, and S2*C*, see "ΔAtad5"). In contrast, Rfc1-, Ctf18-, and Rad17-depletion did not detectably reduce the rate of hPCNA unloading compared to the mock-treated NPE (Figs. 2, *B*–*D*, and S2*C*). Rfc3 loading was significantly reduced in Rfc1-depleted NPE, suggesting that RFC has the strongest binding affinity to closed-circular DNA among all RFC/RLCs. Although these experiments do not exclude the possibility that RFC, Ctf18-RLC, and Rad17-RLC contribute to a minor fraction of hPCNA unloading, they suggest that Atad5-RLC is the major PCNA unloader in *Xenopus* egg extracts.

**Figure 3:**
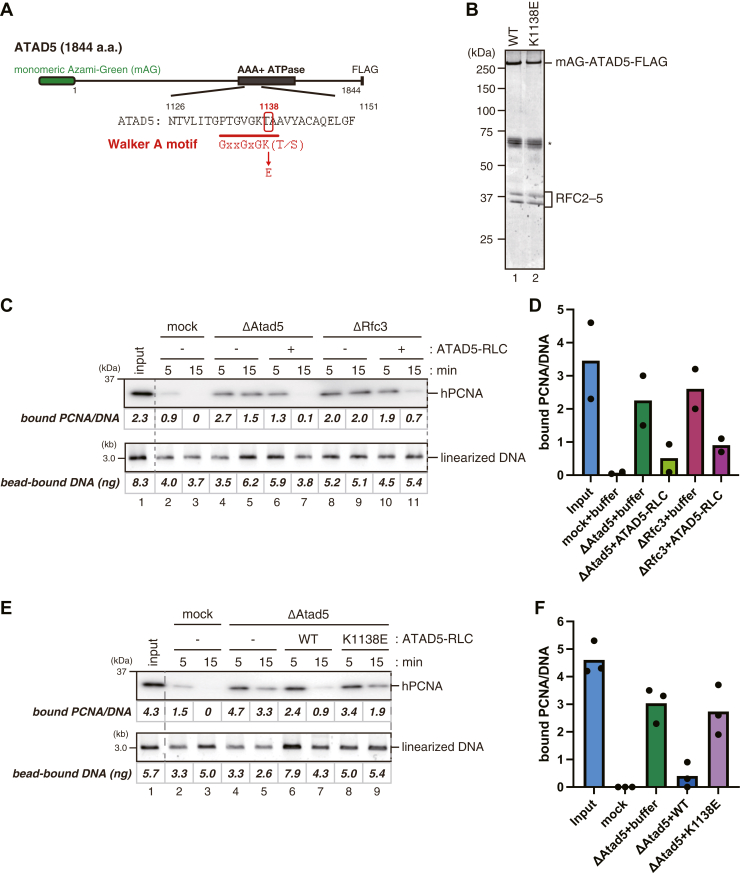
**The Walker A motif of ATAD5 is required for PCNA unloading**. *A*, a schematic diagram of the human ATAD5 expression construct is presented. Monomeric Azami-Green (mAG) and a FLAG epitope were fused at the N- and C-termini, respectively. The Walker A mutant of ATAD5 was made by substituting the conserved lysine 1138 residue with glutamic acid. *B*, 250 ng each of purified wild-type (WT) and the Walker A mutant (K1138E) ATAD5-RLCs were separated by SDS-PAGE and stained with Coomassie brilliant blue R-250. (∗) indicates unrelated proteins co-purified with ATAD5-RLC. *C*, the PCNA unloading assay in mock-treated HSS supplemented with buffer (lanes 2 and 3), Atad5-depleted HSS supplemented with buffer (lanes 4 and 5) or 12 nM ATAD5-RLC (lanes 6 and 7), or Rfc3-depleted HSS supplemented with buffer (lanes 8 and 9) or 12 nM ATAD5-RLC (lanes 10 and 11). An immunoblot of DNA-bound PCNA (*top*) and linearized DNA separated by agarose gel electrophoresis (*bottom*) are presented, along with the estimated numbers of PCNA molecules per DNA and the amounts of DNA. *D*, the average numbers of DNA-bound PCNA per plasmid at the 15-min time points were plotted into a graph. Filled circles represent individual data from two independent experiments, including the one shown in (*C*). *E*, the PCNA unloading assay in mock-treated HSS supplemented with buffer (lanes 2 and 3), or Atad5-depleted HSS supplemented with buffer (lanes 4 and 5), 12 nM ATAD5^WT^-RLC (lanes 6 and 7), or ATAD5^K1138E^-RLC (lanes 8 and 9). An immunoblot of DNA-bound PCNA (*top*) and linearized DNA (*bottom*) are presented, along with the estimated numbers of PCNA molecules per DNA and the amounts of DNA. *F*, the average numbers of DNA-bound PCNA per plasmid at the 15-minute time points were plotted into a graph. Filled circles represent individual data from three independent experiments, including the one shown in (*E*).

To study further the regulation of the PCNA unloading activity of Atad5-RLC, we expressed full-length human ATAD5 with N-terminal monomeric Azami-Green (mAG) and C-terminal FLAG tags in HEK293T cells, either wild-type or a mutant form of the Walker A motif (K1138E), along with smaller RFC2–5 subunits (46), and purified the respective complexes by FLAG affinity chromatography (Fig. 3, *A* and *B*). We found that the high-speed supernatant (HSS) of *Xenopus* eggs containing both cytosolic and nuclear proteins supports efficient PCNA unloading depending on Atad5 (Fig. 3*C*, lanes 1–5) and decided to use HSS as an easy-to-make alternative to NPE. Consistent with the prediction that Atad5-RLC is the major PCNA unloader in *Xenopus* egg extracts, the wild-type ATAD5-RLC restored PCNA unloading not only in Atad5-depleted HSS but also in Rfc3-depleted HSS (Fig. 3, *C* and *D*). In contrast, ATAD5-RLC carrying the K1138E mutation was not capable of restoring PCNA unloading in Atad5-depleted HSS (Fig. 3, *E* and *F*). These results suggest that PCNA unloading by Atad5-RLC depends on its ATP-binding or hydrolysis.

**Figure 4:**
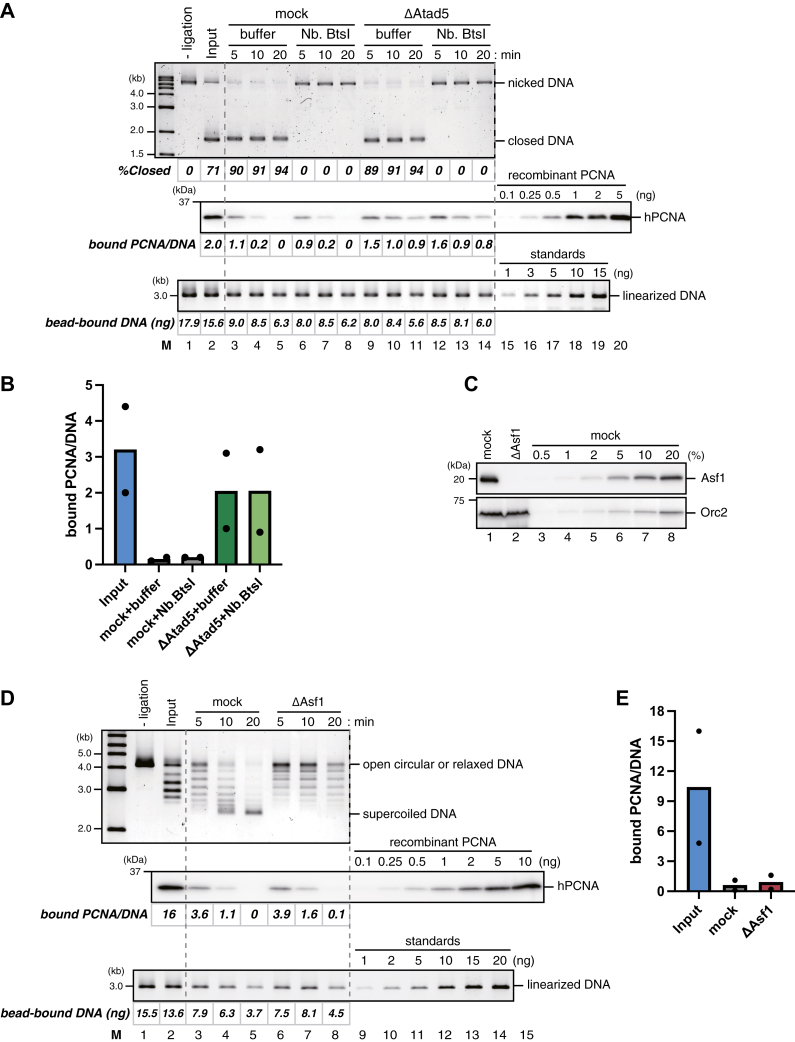
**Single-strand nicks and chromatin assembly do not detectably affect PCNA unloading in *Xenopus* egg extracts**. *A*, the PCNA unloading assay in mock-treated HSS supplemented with buffer (lanes 3–5) or Nb.BtsI (lanes 6–8), or Atad5-depleted HSS supplemented with buffer (lanes 9–11) or Nb.BtsI (lanes 12–14), presented with the input samples before (lane 1) and after nick ligation (lane 2). Untreated DNA separated by agarose gel electrophoresis in the presence of ethidium bromide (*top*), a quantitative immunoblot of DNA-bound PCNA (*middle*), and linearized DNA separated by agarose gel electrophoresis (*bottom*) are shown, along with the estimated numbers of PCNA molecules per DNA and the amounts of DNA. *B*, the average numbers of DNA-bound PCNA per plasmid at the 10-min time points were plotted into a graph. Filled circles represent individual data from two independent experiments, including the one shown in (*A*). *C*, 2 μl each of mock-treated (lane 1) and Asf1-depleted HSS (lane 2) were analyzed by immunoblotting with indicated antibodies, along with a serial dilution series of mock-treated HSS (lanes 3–8). Orc2 serves as a loading control. *D*, the PCNA unloading assay in HSS described in (*C*). Untreated DNA (*top*), a quantitative immunoblot of DNA-bound PCNA (*middle*), and linearized DNA (*bottom*) are presented, along with the estimated numbers of DNA-bound PCNA molecules per plasmid and the amounts of bead-bound DNA. *E*, the average numbers of DNA-bound PCNA per plasmid at the 10-min time points were plotted into a graph. Filled circles represent individual data from two independent experiments, including the one shown in (*D*).

Kubota *et al*. have shown that PCNA unloading occurs only after the completion of Okazaki fragment ligation in yeast (58). To gain further insight into the regulation of PCNA unloading by Atad5-RLC, we next tested whether single-strand nicks or chromatin assembly affects PCNA unloading in *Xenopus* egg extracts. First, we repeated our PCNA unloading assay in the presence of the Nb.BtsI nicking enzyme, which cleaves our DNA substrate at two loci. This enzyme efficiently converted closed-circular DNA into the open-circular form, suggesting continuous nicking in HSS, which supports efficient nick ligation (Fig. 4*A*, top panel). Despite nearly complete conversion of the plasmids into the open form, however, the rate of PCNA unloading was not detectably affected by the presence of the nicking enzyme (Fig. 4*A*, lanes 2–8, and *B*). Also, the observed unloading was largely inhibited by Atad5-depletion (Fig. 4*A*, lanes 9–14, and *B*). These results suggest that Atad5-RLC can unload PCNA regardless of the presence of single-strand nicks in *Xenopus* egg extracts.

Since the completion of DNA synthesis also involves nucleosome assembly, we next tested whether nucleosome assembly affects PCNA unloading. Asf1 is the histone chaperone critical for histone deposition in both replication-coupled (CAF-1-dependent) and replication-independent (HIRA-dependent) pathways, and the depletion of Asf1 effectively inhibits nucleosome assembly in *Xenopus* egg extracts (59). Immunodepletion of Asf1 from HSS indeed prevented the supercoiling of the plasmid substrate, a readout of nucleosome deposition (Fig. 4, *C*–*E*, see “ΔAsf1”). However, it did not significantly affect the rate of PCNA unloading, suggesting that PCNA unloading occurs regardless of chromatin assembly in *Xenopus* egg extracts.

**Figure 5:**
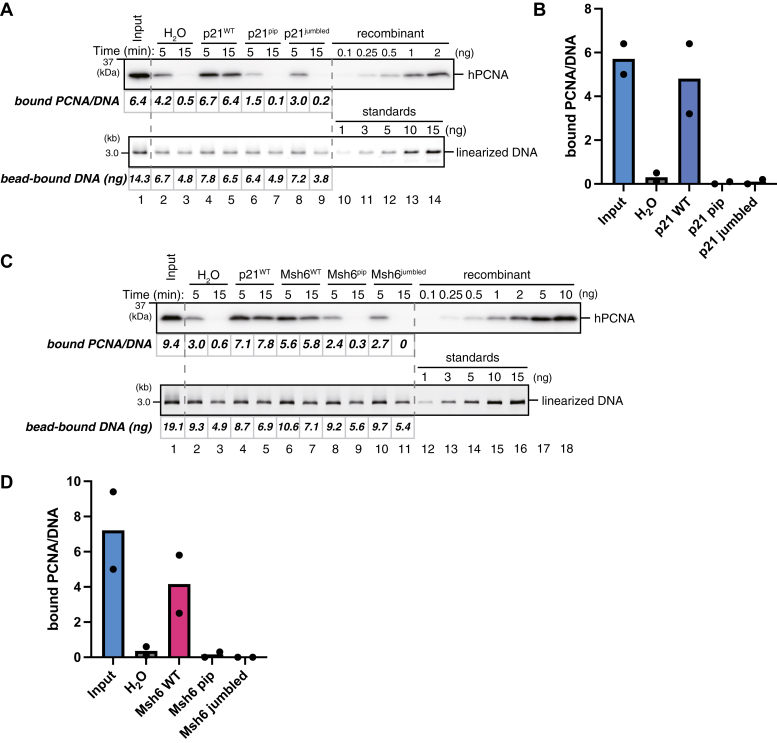
**PCNA-binding peptides inhibit PCNA unloading**. *A*, the PCNA unloading assay in HSS supplemented with the solvent (H_2_O, lanes 2 and 3), wild-type (p21^WT^, lanes 4 and 5), PIP-mutant (p21^pip^, lanes 6 and 7), or sequence-scrambled p21 peptides (p21^jumbled^, lanes 8 and 9). A quantitative immunoblot of DNA-bound PCNA (*top*) and linearized DNA separated by agarose gel electrophoresis (*bottom*) are presented, along with the estimated numbers of PCNA molecules per DNA and the amounts of DNA. *B*, the average numbers of DNA-bound PCNA per plasmid at the 15-min time points were plotted into a graph. Filled circles represent individual data from two independent experiments, including the one shown in (*A*). *C*, the PCNA unloading assay in HSS supplemented with the solvent (H_2_O, lanes 2 and 3), wild-type p21 peptide (p21^WT^, lanes 4 and 5), wild-type (Msh6^WT^, lanes 6 and 7), PIP-mutant (Msh6^pip^, lanes 8 and 9), or sequence-scrambled Msh6 peptides (Msh6^jumbled^, lanes 10 and 11). A quantitative immunoblot of DNA-bound PCNA (*top*) and linearized DNA (*bottom*) are presented, along with the estimated numbers of PCNA molecules per DNA and the amounts of DNA. *D*, the average numbers of DNA-bound PCNA per plasmid at the 15-min time points were plotted into a graph. Filled circles represent individual data from two independent experiments, including the one shown in (*C*).

We have previously shown that the MutSα mismatch sensor complex prevents PCNA unloading to maintain MMR capability (13). Since this reaction largely depends on the PIP motif located on the N-terminus of the Msh6 subunit of MutSα, a plausible scenario is that the binding of the PIP peptide to the hydrophobic pocket on PCNA inhibits PCNA unloading. To test this possibility, we repeated the PCNA unloading assay in the presence of various PCNA-binding peptides. The PIP-peptide of p21 is one of the strongest PCNA-binding peptides, and it indeed effectively prevented PCNA unloading in HSS (Fig. 5, *A* and *B*). Both alanine mutations in the PIP consensus motif and jumbling of the peptide sequence completely inactivated the inhibitory effect of the peptide on PCNA unloading. Likewise, the PIP-peptide from MutSα impeded PCNA unloading, an effect that was neutralized by the PIP mutation and the jumbling of the peptide sequence (Fig. 5, *C* and *D*). These results suggest that PCNA unloading depends on the access of Atad5-RLC to the PIP-binding pocket on PCNA.

## Discussion

By using a method that explicitly distinguishes PCNA unloading from its loading, we show here that Atad5-RLC provides the major PCNA unloading activity in *Xenopus* egg extracts. Thus, Atad5-depletion attenuated the rate of PCNA unloading to the level comparable to Rfc3-depletion, purified ATAD5-RLC restored PCNA unloading in not only Atad5-but also Rfc3-depleted extracts, and Rfc1-, Ctf18-, or Rad17-depletion did not detectably affect the PCNA unloading kinetics (Figs. 2 and 3). Given the fact that Atad5-RLC constitutes only approximately 3% of RFC/RLCs (Fig. 1), it is conceivable that Atad5-RLC is the RFC-family clamp-loader/unloader complex that is specialized for PCNA unloading. Recent structural studies show that the Rfc1 subunit dominates the DNA binding interface over the Rfc2–5 smaller subunits (60, 61, 62), and the Rad24 subunit of Rad24-RLC directs its binding to the double-stranded region at a 5′ junction of single-stranded and double-stranded DNA in an arrangement opposite to RFC (36, 37). Therefore, it is highly likely that the largest subunits critically regulate the DNA binding specificity of RFC/RLCs, thereby restricting the situation in which each complex functions. Since almost all Rfc3 and Rfc5 are included in the RFC/RLC complexes (Figs. 1 and S1*A*), the large subunits may limit the PCNA loading/unloading activity residing in the smaller subunits.

A key regulation of PCNA unloading is to distinguish PCNA molecules that are no longer used from those in use. Since the ligation of Okazaki fragments is an essential prerequisite for PCNA unloading in yeast cells (58), the regulation of PCNA unloading is likely linked to Okazaki fragment maturation. The trimeric nature of PCNA enables it to function as a molecular toolbelt, which sequentially recruits Pol δ, Fen1, and Lig1 to coordinate flap processing and nick ligation during Okazaki fragment maturation (63, 64, 65, 66). The inactivation of Lig1 may compromise the disassembly of the PCNA toolbelt complex and prevent the access of the PCNA unloader to PCNA. Consistent with this prediction, Kang *et al*. showed that Okazaki fragment maturation factors inhibit PCNA unloading by ATAD5-RLC in a purified system (47), and we found that PCNA-interacting peptides, but not continuous nicking of DNA, strongly impede PCNA unloading in a physiological condition recapitulated in *Xenopus* egg extracts (Figs. 4 and 5). Therefore, PCNA interactors can indeed prevent PCNA unloading. Interestingly, a biochemical study showed that Lig1 remains bound onto DNA along with PCNA even after the ligation of nicks (66). It is to be determined whether PCNA must be completely free of its interactors to be unloaded or whether some PCNA interactors are compatible with PCNA unloading.

The above findings provide a framework as to how PCNA-mediated reactions are regulated: PCNA interactors prevent its unloading to secure the PCNA clamps that are in use. The completion of DNA synthesis, repair, and Okazaki fragment maturation liberates PCNA from its interactors, allowing the access of the PCNA unloader to DNA-bound PCNA. In addition, PCNA unloading can be inhibited by supplying PIP peptides around DNA-bound PCNA. PCNA is a strand-discrimination marker for eukaryotic MMR (12), and ongoing MMR inhibits PCNA unloading to maintain the strand-discrimination capability (13). The MMR system likely does so by loading multiple MutSα onto a mismatch site and providing a high concentration of the PIP peptides, each of which is located at the N-terminus of a long and flexible linker on the Msh6 subunit (67).

## Experimental procedures

### Preparation of *Xenopus* egg extracts

*X. laevis* was purchased from Kato-S-Science and handled according to the animal experimental regulations at Kyushu University. NPE and HSS were prepared as described previously (68, 69).

### Cloning

Symbols of human and *Xenopus* genes and proteins conformed to the nomenclature guidelines of HGNC (https://www.genenames.org/about/guidelines/) and Xenbase (https://www.xenbase.org/entry/static/gene/geneNomenclature.jsp).

Cloning of the *X. laevis rfc3* gene was performed as follows: The *rfc3* gene was amplified from *Xenopus* egg cDNA by two-step PCR using primers listed in Supporting Table S1 and cloned into pDONR201 (Thermo Fisher Scientific) by the Gateway BP reaction, resulting in pDONR-xRFC3. For protein expression in *Escherichia coli*, the *rfc3* gene on pDONR-xRFC3 was transferred into pET-HSD, an in-house Gateway destination vector carrying a T7 promoter and an N-terminal His_6_-tag, by the Gateway LR reaction.

Cloning of the human *ATAD5* gene was performed as follows: The *ATAD5* gene was amplified from the HeLa cDNA library with a FLAG-tag sequence fused to the C-terminus of ATAD5 by two-step PCR using primers listed in Supporting Table S1, digested with BamHI (New England Biolabs, #R3136) and SbfI (New England Biolabs, #R3642), and ligated with a BamHI/SbfI-digested vector fragment that had been amplified by PCR from pCSII-EF-mAG-TEV-6His-Claspin-3Flag (70) using primers listed in Supporting Table S1, resulting in pCSII-EF-mAG-hATAD5-FLAG. The Walker A motif mutant of hATAD5 (hATAD5-K1138E) was constructed by two-step PCR using primers listed in Supporting Table S1. The amplified fragment was digested with BamHI and HpaI (New England Biolabs, #R0105) and inserted between the BamHI and HpaI sites in pCSII-EF-mAG-hATAD5-FLAG, resulting in pCSII-EF-mAG-hATAD5-K1138E-FLAG.

### Protein expression and purification

Expression and purification of wild-type and the Walker A mutant (K1138E) of ATAD5-RLC, human PCNA, and human RFC were carried out as described previously (13, 71).

Expression and purification of the His_6_-tagged *Xenopus* Rfc3 protein were performed as follows: Recombinant protein expression was induced in *E.coli* BL21-CodonPlus (DE3)-RIPL cells transformed with pET-HSD-xRFC3 by the addition of 0.5 mM Isopropyl β-D-1-thiogalactopyranoside (IPTG) in Terrific broth for 2 h at 37 °C. Cells were harvested, lysed with 1 mg/ml lysozyme, sonicated in buffer S (50 mM Na-phosphate pH 8.0, 300 mM NaCl, 5% glycerol, 0.1% Triton X-100, 2.5 mM 2-mercaptoethanol, 1 mM phenylmethylsulfonyl fluoride [PMSF]), and centrifuged at 20,400*g* for 10 min. Inclusion bodies containing the Rfc3 protein were resuspended in buffer S, sonicated, and centrifuged again at 20,400*g* for 5 min, and the procedure was repeated twice. The Rfc3 protein was extracted from purified inclusion bodies with Laemmli’s SDS sample buffer.

### Immunological methods

Production and usage of antibodies against *Xenopus* Rfc3 were described previously (13). The mouse monoclonal antibody against human PCNA (MBL International Corporation, #MH-12–3) is commercially available. The rabbit antiserum against *Xenopus* Orc2 was a kind gift from Dr Johannes Walter. The rabbit Ctf18, Rad17, Rfc5, Ctf8, and Asf1 antibodies were raised against peptides NH_2_-CINEEFGENDSEILENDDNA-COOH, corresponding to residues 269 to 287 of Ctf18, NH_2_-CAAQAIMEDEELKIEEYDSD-COOH, corresponding to residues 269 to 287 of Rad17, NH_2_-CEHVVKEERVDISPDGMK-COOH, corresponding to residues 182 to 198 of Rfc5, NH_2_-CKIIFKTRPKPIITNVPKKV-COOH, corresponding to residues 103 to 121 of Ctf8, NH_2_-APSKGLAAALNTLPENSMDC-COOH, corresponding to residues 180 to 199 of Asf1, respectively. All antibodies except anti-Ctf8 and anti-Asf1 were affinity-purified using corresponding antigens. Polyclonal antibodies against *Xenopus* Atad5 and Rfc1 were raised in rabbits against the following polypeptides: Atad5: N-terminal 105 amino acids of Atad5 with a 6-histidine tag at the C-terminus (MVGILAMSASLEEYGCQPCKKSRKDEEAPIKTITNYFSPVSKNTEKVLSSPRSNNIADYFKQNSPINEKKQTSKAENAAIQQDTPQAAVADSSAASGKPSKCRKR); Rfc1: N-terminal 100 amino acids of Rfc1 with a 6-histidine tag at the C-terminus (MDIRNFFGVKPVAKKHGTEKTDIKEKKKSPEAKKKPKDSKVKSPSS DDSLKGMNVKKKKRIIYDSDAEEESPPVKKAKKPSEKSPPLPRPHKIRKPDPVV). The specificity of all the antibodies has been validated by immunoprecipitation and immunoblotting using recombinant proteins and *Xenopus* egg extracts.

For immunoblotting, the Orc2 antiserum was used at a dilution of 1:10,000, and the Rfc1, Atad5, Ctf8, and Asf1 antisera were used at 1:5000 dilutions. For immunoblotting of Ctf18, Rad17, Rfc3, and Rfc5, affinity-purified antibodies were used at 0.5 μg/ml. Alexa fluor 647 conjugated Goat anti-rabbit IgG (H + L) antibodies (Jackson ImmunoResearch, #111–605–144), Goat anti-mouse IgG (H + L) antibodies (Jackson ImmunoResearch, #115–035–146), and Monoclonal Mouse anti-rabbit IgG specific to the light chain (Jackson ImmunoResearch, #211–602–171) were used at 1:10,000 dilutions as the secondary antibodies.

For immunodepletion of Rfc1, Atad5, or Asf1, 3 vol of an antiserum was bound to 1 vol of recombinant Protein A Sepharose Fast Flow (PAS; Cytiva, #17127902) at 4 °C overnight. For Ctf18, Rad17, or Rfc3 depletion, 5 μg of purified IgG was bound to 1 μl PAS. To deplete extracts, 0.2 vol of the antibody-coupled PAS beads were incubated with 1 vol of NPE or HSS at 4 °C for 1 h, and the procedure was repeated twice. In most cases, we depleted 20∼60 μl of extracts for an experiment. For quadruple depletion of Rfc1, Ctf18, Atad5, and Rad17 from NPE, antibody-coupled beads were prepared separately as described above, 2 μl each of IgG beads were combined, and 8 μl of the combined IgG beads were incubated in 10 μl of NPE that had been diluted to 50 μl (5-fold) with Egg lysis buffer salts (ELB-salts: 10 mM Hepes-KOH pH 7.7, 2.5 mM MgCl_2_, 50 mM KCl) at 4 °C for 1 h, and the procedure was repeated twice.

### Quantification of RFC/RLCs in *Xenopus* egg extracts

The concentration of RFC and other RLCs was estimated from the amount of Rfc3 co-precipitated with the largest subunits. For IP of Rfc1 or Atad5, 1.5 vol of an antiserum was bound to 1 vol of PAS, and for IP of Rfc3, Ctf18, or Rad17, 15 μg of affinity-purified antibodies were bound to 1 μl PAS at 4 °C overnight. NPE was diluted 10-fold with ELB-salts and clarified by centrifugation at 20,400*g* for 10 min to remove insoluble debris. 10 μl each of antibody-coupled beads were incubated with 100 μl of diluted NPE at 4 °C for 1h, washed three times with ELB-salts, and bound proteins were eluted with 40 μl of Laemmli’s SDS sample buffer. IP samples were separated by SDS-PAGE, alongside a dilution series of recombinant Rfc3, transferred onto polyvinylidene fluoride membranes, and probed with antibodies against Rfc3 and other subunits. The amounts of Rfc3 in the IP fractions were calculated using the dilution series of recombinant Rfc3 as a standard.

### Preparation of substrates for the *in vitro* PCNA-loading assay

*In vitro* synthesis of plasmids carrying a site-specific biotin modification was performed as described previously (13). Briefly, an oligonucleotide carrying a biotin-dT modification was annealed on a 3-kb single-stranded phagemid DNA, and the complementary DNA strand was synthesized by in-house-purified T7 DNA polymerase. After ligation of nicks by T4 DNA ligase (Nippon gene, #311–00404), covalently closed DNA molecules were purified by cesium chloride density gradient ultra-centrifugation. To make a PCNA loading site, a locus-specific nick was introduced by Nt.BbvCI (New England Biolabs, #R0632). The DNA was bound onto Sepharose beads at a ratio of 100 ng per 1 μl, following the procedure described previously (72).

### *In vitro* PCNA-loading assay

The *in vitro* PCNA-loading assay was performed as described previously, with some modifications (13). Briefly, DNA beads were washed three times with mHBS buffer (10 mM Hepes-NaOH pH 7.5, 0.05% Tween-20, 10 mM MgCl_2_, 200 μM ethylenediaminetetraacetic acid [EDTA], 150 mM NaCl) and incubated in 2 vol of mHBS containing 50 mM phosphocreatine (PC), 25 μg/ml creatine phosphokinase (CPK), 2 mM adenosine triphosphate (ATP), 400 μM dithiothreitol (DTT), 145 ng/μl hPCNA, and 2.2 ng/μl RFC at 32 °C for 15 min. The PCNA-DNA complex was washed three times with mHBS and incubated in 4 vol of ligation buffer (50 mM Tris-HCl pH 7.9, 10 mM, MgCl_2_, 20 mM DTT, 1 mM ATP) containing 25 units/μl T4 DNA ligase at 32 °C for 5 min, washed three times with mHBS, once with ELB-salts containing 1 M KCl, and then once with ELB-salts.

### The PCNA unloading assay in *Xenopus* egg extracts

*Xenopus* egg extracts (HSS and NPE) were supplemented with 2 mM ATP, 20 mM PC, 5 μg/ml CPK, and pre-incubated at 22 °C for 5 min. The PCNA-DNA complex was incubated in the extract at the concentration of 20 ng/μl with respect to immobilized DNA at 22 °C. At appropriate time points, the mixture was quickly diluted with 200 μl of ELB-salts containing 0.2% Triton X-100, overlayed onto 200 μl of ELB-salts containing 0.5 M Sucrose and centrifuged at 12,700*g* for 1 min at 4 °C in a TMS-21 swinging bucket rotor (Tomy Seiko, #0621650333). The beads were washed once with 200 μl of ELB-salts and resuspended in an appropriate volume of ELB-salts buffer. DNA beads were then split into two aliquots to quantify the amount of bead-bound DNA and DNA-loaded PCNA. To quantify DNA, an aliquot of the beads was mixed with 100 μl of stop buffer (1% sodium dodecyl sulfate [SDS] and 20 mM EDTA) and treated with 50 μg/ml Proteinase K (Nacalai Tesque, #29442–14) at 37 °C for 1 h. DNA was then purified by phenol/chloroform extraction and ethanol precipitation and resuspended in TE buffer (10 mM Tris-HCl pH 7.4, 1 mM EDTA) containing 10 μg/ml RNase A. DNA samples were digested with XmnI (New England Biolabs, #R0194) and analyzed by 0.8% agarose gel electrophoresis followed by staining with SYBR Gold nucleic acid gel stain (Thermo Fisher Scientific, #S11494). Fluorescent signals were detected using the Amersham Typhoon scanner five system (Cytiva) and processed using the ImageQuant TL software (Cytiva). To evaluate the PCNA amount, another aliquot of the beads was mixed with Laemmli’s SDS sample buffer, and DNA-bound proteins were analyzed by SDS-PAGE followed by immunoblotting. The p21 and Msh6 peptides (p21 wild-type, NH_2_-KRRQTSMTDFYHSKRRLIFS-COOH; p21 pip, NH_2_-KRRATSATDAAHSKRRLIFS-COOH; p21 jumbled, NH_2_-QDKTRYFHRTMSRSKSIRLF-COOH; Msh6 wild-type, NH_2_-MSKQKTLFSFFTKSPPVSSS-COOH; Msh6 pip, NH_2_-MSKAKTLFSAATKSPPVSSS-COOH; Msh6 jumbled, NH_2_-SVSSFTPKLQSFKSPTSFKM-COOH) (73) were synthesized by Eurofins Genomics, and added to HSS at the final concentration of 1 mg/ml. To introduce nicks on plasmids, Nb.BtsI (New England Biolabs, #R0707) was added to HSS at the concentration of 0.5 units/μl. For heat-inactivation, HSS was boiled at 95 °C for 5 min, centrifuged at 20,400*g* for 10 min at 4 °C, and the supernatant was recovered as heat-inactivated HSS.

### Quantification of DNA-bound PCNA

The amount of bead-bound DNA and DNA-bound PCNA was quantified using known amounts of XmnI-digested DNA and recombinant PCNA as standards, respectively. To calculate DNA-bound PCNA per plasmid, the estimated number of PCNA-trimer molecules was normalized to that of bead-bound DNA molecules.

## Data availability

All data from this study are included in the manuscript and supporting information. Unprocessed raw data and DNA sequences underlying this article will be shared on reasonable request to the corresponding authors.

## Supporting information

This article contains supporting information. Supporting figures include co-depletion of the small subunits with the large subunits in NPE, the recombinant protein used for quantification (Fig. S1), the stability of DNA-bound hPCNA in heat-inactivated *Xenopus* egg extracts, and a graph of the time course experiments shown in Figure 2*C* (Fig. S2). Supporting Table S1 summarizes the oligonucleotide primers used in this study.

## Conflict of interest

The authors declare no competing interest.
