# Supplementary material for: The Atad5 RFC-like complex is the major unloader of proliferating cell nuclear antigen in Xenopus egg extracts
Source: J Biol Chem. 2023 Dec 21;300(1):105588. doi: 10.1016/j.jbc.2023.105588 (PMC10827553; doi:10.1016/j.jbc.2023.105588)
Supplement: Supporting information [file mmc1.pdf]

Figure S1

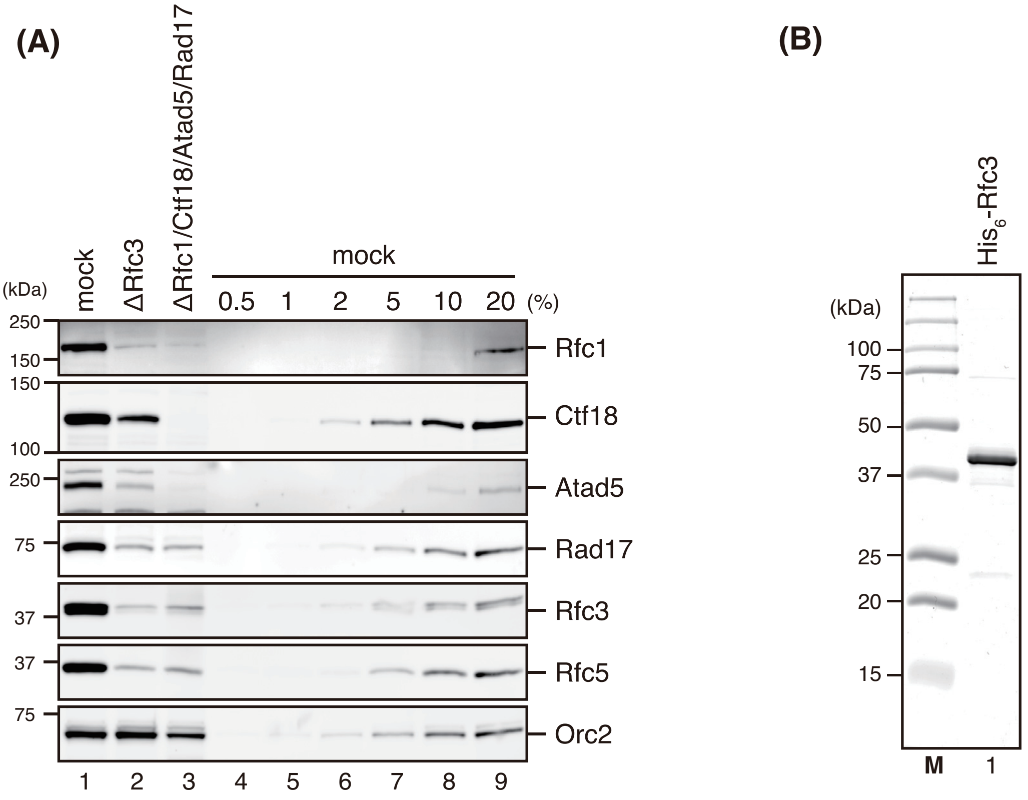

(A)
(B)
mock
ΔRfc3
ΔRfc1/Ctf18/Atad5/Rad17
mock
0.5
1
2
5
10
20
(%)
(kDa)
250
150
150
100
250
75
37
37
75
Rfc1
Ctf18
Atad5
Rad17
Rfc3
Rfc5
Orc2
1 2 3 4 5 6 7 8 9
His6-Rfc3
(kDa)
100
75
50
37
25
20
15
M
1

Supporting Figure S1: Most of Rfc3 and Rfc5 are included in RFC and RLCs in *Xenopus* egg extracts

(*A*) 0.25 µL each of mock-treated (lane 1), Rfc3-depleted (lane 2), and Rfc1/Ctf18/Atad5/Rad17-depleted NPE (lane 3) were analyzed by immunoblotting with the indicated antibodies alongside a serial dilution series of mock-treated NPE (lanes 4–9). Orc2 serves as a loading control. Quadruple depletion of Rfc1, Ctf18, Atad5, and Rad17 removed more than 90% of Rfc3 and Rfc5, suggesting that most of the small subunits in *Xenopus* egg extracts are included in RFC and RLCs.

(*B*) 2.3 µg of recombinant His_6-Rfc3 purified from *E. coli* was separated by SDS-PAGE and stained with Coomassie brilliant blue R-250.

Figure S2

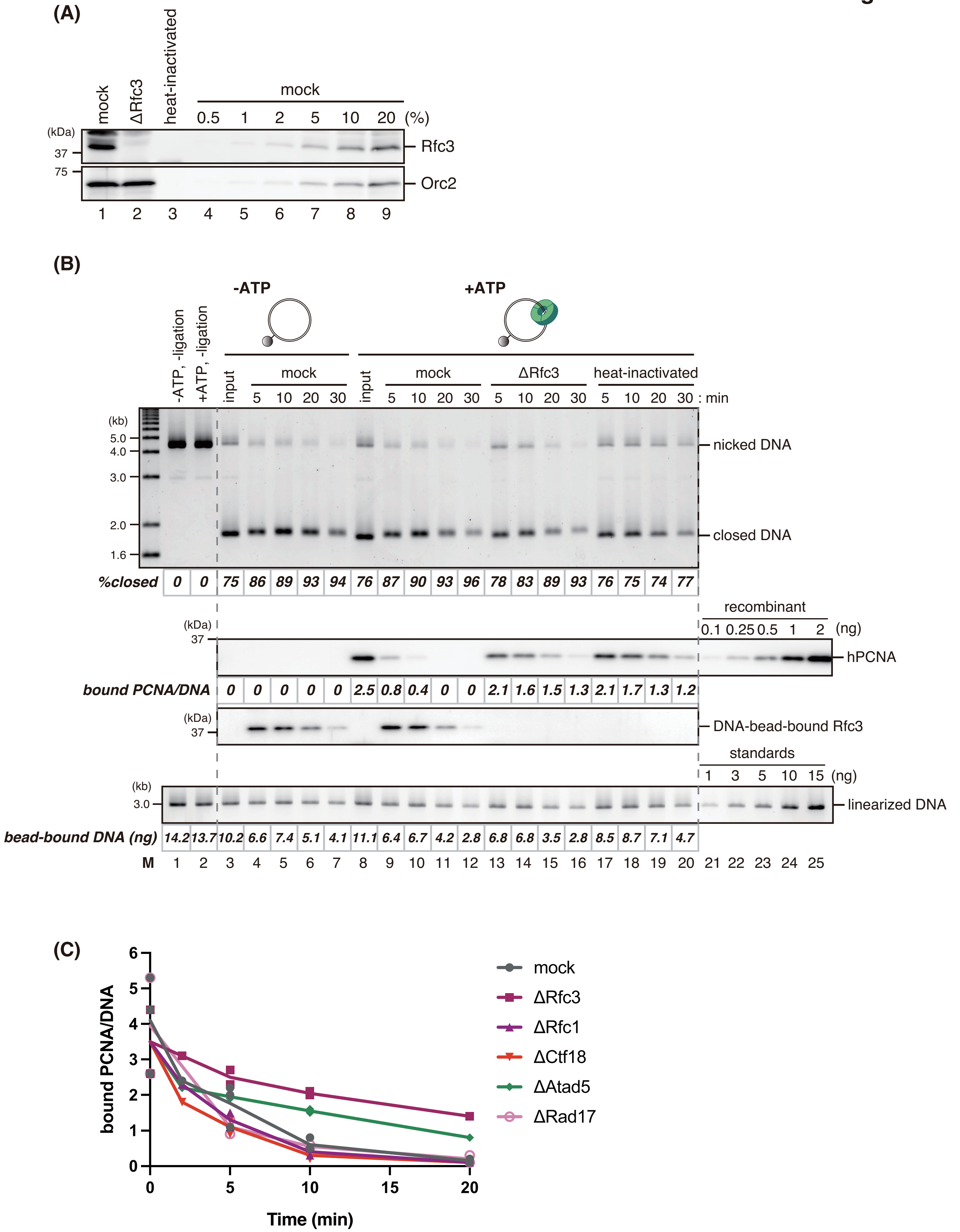
(A)
mock
ΔRfc3
heat-inactivated
mock
0.5 1 2 5 10 20 (%)
(kDa)
37
75
Rfc3
Orc2
1 2 3 4 5 6 7 8 9
(B)
-ATP
+ATP
-ATP, -ligation
+ATP, -ligation
input
mock
ΔRfc3
heat-inactivated
5 10 20 30 : min
(kb)
5.0
4.0
3.0
2.0
1.6
nicked DNA
closed DNA
%closed
0 0 75 86 89 93 94 76 87 90 93 96 78 83 89 93 76 75 74 77
recombinant
0.1 0.25 0.5 1 2 (ng)
(kDa)
37
hPCNA
bound PCNA/DNA
0 0 0 0 0 2.5 0.8 0.4 0 0 2.1 1.6 1.5 1.3 2.1 1.7 1.3 1.2
DNA-bead-bound Rfc3
standards
1 3 5 10 15 (ng)
linearized DNA
bead-bound DNA (ng)
14.2 13.7 10.2 6.6 7.4 5.1 4.1 11.1 6.4 6.7 4.2 2.8 6.8 6.8 3.5 2.8 8.5 8.7 7.1 4.7
M 1 2 3 4 5 6 7 8 9 10 11 12 13 14 15 16 17 18 19 20 21 22 23 24 25
(C)
bound PCNA/DNA
Time (min)
0 5 10 15 20
0 1 2 3 4 5 6
mock
ΔRfc3
ΔRfc1
ΔCtf18
ΔAtad5
ΔRad17

Supporting Figure S2: Spontaneous dissociation of PCNA from DNA in heat-inactivated *Xenopus* egg extracts

(*A*) 2 µL each of mock-treated (lane 1), Rfc3-depleted (lane 2), and heat-inactivated HSS (lane 3), from which heat-denatured insoluble proteins had been removed, were analyzed by immunoblotting with the indicated antibodies alongside a serial dilution series of mock-treated HSS (lanes 4–9). Orc2 serves as a loading control.

(*B*) The *in vitro* PCNA loading assay in the absence (lanes 1 and 3–7) or the presence of ATP (lanes 2 and 8–20) in mock-treated (lanes 4–7 and 9–12), Rfc3-depleted (lanes 13–16) or heat-inactivated HSS (lanes 17–20). Untreated DNA separated by agarose gel electrophoresis in the presence of ethidium bromide (*top*), a quantitative immunoblot of hPCNA and Rfc3 in the bead-bound fractions (*middle*), and linearized DNA separated by agarose gel electrophoresis (*bottom*) are presented along with the percentage of covalently-closed plasmids, the estimated numbers of DNA-bound PCNA molecules per plasmid, and the amounts of DNA. PCNA loaded onto immobilized DNA was dissociated in Rfc3-depleted and heat-inactivated HSS with similar kinetics, suggesting that the observed PCNA dissociation in Rfc3-depleted extracts reflects the spontaneous dissociation of PCNA from DNA.

(*C*) The numbers of DNA-bound PCNA per plasmid, calculated from two independent experiments, including the one shown in Fig. 2C, were plotted into a graph, with lines connecting the mean values. It should be noted that samples were taken at 0, 2, 5, and 10 minutes in a replicate experiment and at 0, 5, 10, and 20 minutes in the other replicate experiment for ΔRfc3, ΔRfc1, ΔCtf18, and ΔAtad5, and at 0, 5, 10, and 20 minutes in both two replicates for ΔRad17. Only the mock control samples were triplicated accordingly, with the sampling time points at 0, 2, 5, and 10 minutes in a replicate experiment and at 0, 5, 10, and 20 minutes in the other two replicate experiments.

Supproting Table S1: oligonucleotides used in this study

Name	Sequence (5'-3')	Application	Genes
1116	AAAGCAGGCTCCACCATGAGTTTGTGGGTTGATAAGTATCGACC	Cloning (Forward)	*Xenopus rfc3* with a partial Gateway attB sequence
1117	ACAAGAAAGCTGGGTCTCAAAACATCATCGCTTCTAGCCCATCC	Cloning (Reverse)	*Xenopus rfc3* with a partial Gateway attB sequence
344	GGGGACAAGTTTGTACAAAAAAGCAGGCTCCAC	Cloning (Forward)	Gateway attB
345	GGGGACCACTTTGTACAAGAAAGCTGGGTC	Cloning (Reverse)	Gateway attB
ATAD5-1F-BamHI	GGAAGGATCCATGGTGGGGGTCCTGGCCATGGCGGC	Cloning (Forward) and Mutagenesis (Forward, Fragment 1)	*human ATAD5*
ATAD5-5535R	TTAAGGGAAGTCAGCTGCCAAAGTATTTACAGTCTC	Cloning (Reverse)	*human ATAD5*
ATAD5-5532R-FL-SbfI	GGAACCTGCAGGTTACTTGTCATCGTCATCCTTGTAGTCTCGAGGGAAGTCAGCTGCCAAAGTATTTAC	Fusion of a FLAG-tag and a SbfI site to ATAD5 (Reverse)	*human ATAD5* with a FLAG-tag sequence and a SbfI site
pCSII-EF-MCS-F	GGAACCTGCAGGGCGGCCAACATCGAGGGATCAAGCTTATCGAT	Cloning (Forward)	pCSII-EF plasmid backbone
pCSII-EF-mAG-R	GGAAGGATCCATGGTGGTGATGGTGGTGGC	Cloning (Reverse)	pCSII-EF plasmid backbone with mAG, TEV cleavage site, and His_6 sequences
ATAD5-K1138E-R	GCAGCAGTTTCTCCCACTCCTGTTGGCCCTG	Mutagenesis (Reverse, Fragment 1)	*human ATAD5*
ATAD5-K1138E-F	GGAGTGGGAGAAACTGCTGCAGTGTATGCTTG	Mutagenesis (Forward, Fragment 2)	*human ATAD5*
ATAD5-4163R-HpaI	GCAGTTAACAAGGTTACAAAG	Mutagenesis (Reverse, Fragment 2)	*human ATAD5*
